# Health insurance literacy in Israel: gaps between knowledge and use in a universal healthcare system

**DOI:** 10.1186/s13584-026-00759-y

**Published:** 2026-04-16

**Authors:** Reut Ron, Paula Feder-Bubis, Moriah E. Ellen

**Affiliations:** 1 Department of Health Policy and Management, Ben-Gurion University of the Negev, P. O. B. 653, Beer-Sheva, 8410501 Israel; 2 Assuta Health Research Institute, HaBarzel 20, Tel Aviv, Israel; 3 Department of Health Policy Management and Evaluation, University of Toronto Dalla Lana School of Public Health, Toronto, ON Canada

**Keywords:** Demographic disparities, Health insurance literacy, Healthcare equity, Israel, Multidimensional assessment

## Abstract

**Background:**

Health Insurance Literacy (HIL) is critical for navigating healthcare systems and making informed insurance decisions. In Israel, which has a universal system with voluntary insurance, disparities in HIL may contribute to inequitable access. This study assessed HIL among diverse Israeli groups using a validated, culturally adapted, multidimensional tool.

**Methods:**

A nationally stratified sample of Hebrew-speaking and Arabic-speaking Israeli adults (1,012) completed an online cross-sectional survey in September 2024. A 75-item questionnaire combined an adapted Health Insurance Literacy Measure (HILM), self-reported assessments, and objective knowledge tests. Analyses (ANOVA, regression) examined relationships between demographics and HIL dimensions.

**Results:**

HIL levels were moderate (mean HILM score: 2.39/4), with significant disparities across demographic groups. Non-Ultra-Orthodox Israeli-born Jews showed the highest literacy, while Arab and foreign-born Jews displayed lower comprehension. Key predictors included income, self-reported health, and insurance type (*p* < 0.001), while education was weaker than expected.

**Conclusions:**

Findings highlight systemic and cultural barriers to HIL in Israel and the need for targeted educational interventions, simplified communication tools, and culturally tailored programs. This study offers a framework for evaluating HIL in publicly funded systems and insights for other multicultural settings.

**Supplementary Information:**

The online version contains supplementary material available at 10.1186/s13584-026-00759-y.

## Background

Health Insurance Literacy (HIL) refers to an individual’s ability to acquire, comprehend, and utilize health insurance-related information, to make informed decisions about healthcare coverage and services. This multidimensional concept includes cognitive understanding of insurance terminology, navigation of administrative processes, and engagement with health insurance systems [1].

HIL is increasingly recognized as a key factor in healthcare accessibility, especially in complex systems where low literacy can deepen inequities [2]. Studies globally show individuals often struggle with basic insurance concepts, plan selection, and system navigation [3, 4], underscoring the need for reliable, context-specific assessment tools.

The Health Insurance Literacy Measure (HILM) is a validated multidimensional instrument that assesses individuals’ ability to understand, choose, and use health insurance, including conceptual knowledge, confidence, and practical navigation skills, and has been widely adopted in previous research [1]. However, its application in multicultural contexts with publicly funded health insurance systems, such as Israel, presents limitations. Recent adaptations of the HILM underscore the importance of culturally sensitive modifications to ensure measurement relevance and accuracy [5, 6].

Israel’s health system combines universal public coverage with optional supplementary and private insurance. Under the National Health Insurance Law, all residents receive a standard care package through one of four health plans. Many supplement this with health plan-based plans or private insurance, while an approximately one fifth of the population relies solely on the basic national coverage, with higher rates in certain demographic groups [7].

However, the coexistence of public, supplementary, and private insurance, which often provide overlapping benefits rather than distinct layers has raised concerns among policy experts about the public’s ability to make informed insurance decisions. Prior work has documented substantial gaps in knowledge and understanding of insurance concepts among Israeli residents [8]. Moreover, leading policy discussions have emphasized the need to adapt the National Health Insurance Law to the realities of the 21 st century, including rising costs and increasing consumer responsibility [9], and to address challenges stemming from the rapid expansion of private insurance [10].

Disparities in HIL levels are evident, with over 31% of Israeli adults exhibiting suboptimal health literacy, and pronounced differences observed across socioeconomic and cultural groups [11, 12]. Research highlights lower health insurance comprehension among Arab populations and foreign-born communities, particularly those from the former Soviet Union and Ethiopia, compared to Israeli-born Jews populations [13, 14].

Research on literacy more broadly, including general literacy, health literacy, and financial literacy, consistently demonstrates systematic disparities across socioeconomic, cultural, and population groups [15, 16]. Lower literacy levels have been documented among individuals with lower income and education, minority populations, immigrants, and groups facing structural barriers to information access and institutional trust [17, 18]. In Israel, national surveys on health literacy and functional literacy similarly reveal persistent gaps between Jewish and Arab populations, between Israeli-born and immigrant groups, and across levels of socioeconomic status [14, 19].

Importantly, while these disparities appear across multiple literacy domains, previous research suggests that insurance-related literacy may exhibit distinct patterns [2]. HIL requires not only comprehension skills, but also navigation of complex administrative systems, interpretation of legal and financial terminology, and experiential engagement with health services [20]. As such, the extent to which disparities in HIL mirror or diverge from patterns observed in other literacy domains remains an open empirical question, particularly in universal health coverage systems such as Israel’s.

We identified three key gaps in the existing literature. First, there is a lack of nationally representative data on HIL in Israel. Second, previous studies have not employed a culturally adapted, multidimensional tool capable of assessing different domains of HIL. Third, limited evidence exists regarding sector-specific disparities across major demographic groups in a universal health coverage system. Addressing these gaps is essential for understanding how individuals navigate insurance complexity and where targeted interventions may be needed.

Building on these gaps, the aim of this study is to evaluate HIL among the Israeli population using an innovative, validated questionnaire. By assessing key dimensions of HIL, including knowledge and understanding, confidence and use, the research seeks to provide in-depth analysis of the Israeli public’s grasp of health insurance. This study makes a distinct contribution to the literature by providing the first comprehensive, nationally stratified assessment of HIL in Israel using a multidimensional framework, while explicitly examining the gap between conceptual knowledge and behavioral competencies in a universal healthcare system.

## Methods

The study utilized a nationally stratified sample of Israeli adults recruited through an online panel, designed to approximate key demographic distributions, to generate nuanced insights into HIL levels across different demographic groups, particularly among populations shown in prior research to have lower literacy, such as Arab communities and foreign-born Jews populations, and inform targeted educational interventions delivered by health plans, the Ministry of Health, and other relevant stakeholders.

In September 2024, an online survey was conducted to assess HIL levels among the Israeli population, as a cross-sectional study. Data collection was conducted by Midgam Research and Consulting, a leading Israeli research firm. Participants were sampled from iPanel national probability-based online panel. Stratification was based on age, gender, geographic region, and population sector. Within each stratum, participants were randomly selected using computerized probability sampling to achieve proportional representation of the Israeli adult population.

The survey was administered through Midgam’s national online panel. As is common in probability-based online panels in Israel, the research firm does not provide information on the total number of panel members exposed to the survey invitation. Therefore, a conventional response rate could not be calculated. This limitation is inherent to panel-based survey methodologies and should be considered when interpreting the findings. Of the participants who entered the survey environment and met the quota-based eligibility criteria, 1,012 respondents completed the full questionnaire. The completion rate among eligible participants was high, and all demographic quotas were achieved.

A 75-item questionnaire was developed based on an extensive literature review and adapted from the validated HILM [1]. It incorporated six sections: (1) HILM (21 items, 1–4 scale), (2) self-perceived understanding of key insurance terms (11 items), (3) true/false knowledge questions (23 items, scored 0–23), (4) overall self-rated HIL (1–10 scale), (5) insurance use and purchasing behavior (3 items), and (6) demographic characteristics (16 items). The questionnaire was culturally adapted and translated into Arabic and Russian using back-translation. For transparency and replication purposes, the complete English version of the questionnaire is available in the Supplementary Material.

The questionnaire underwent a multi-stage validation process. Content validity was reviewed by a panel of health policy experts. The translated versions underwent forward–backward translation followed by reconciliation. To ensure linguistic clarity and cultural appropriateness, several Hebrew, Arabic, and Russian-speaking reviewers examined the translated items and provided feedback on wording and comprehension. Revisions were made to improve clarity and address cultural nuances.

A pilot study was conducted with 45 participants from diverse demographic backgrounds, which included tracking completion time and assessing respondent burden. Based on the pilot findings, several items that showed limited variability or contributed to respondent fatigue were removed. An exploratory factor analysis (EFA) was conducted to assess and confirm the dimensional structure of the adapted HILM components. HIL total score was calculated as the mean of all sections, scored on a 1-to-4 scale. These scoring methods allowed for an overall assessment of HIL across multiple dimensions and facilitated detailed analyses and broader categorizations.

The analytic strategy was aligned with the structure of the questionnaire, with each major component analyzed as a separate outcome in addition to a combined index. Accordingly, five dependent outcomes were examined: (1) the HILM total score, (2) the ‘understanding of insurance concepts’ score, (3) the objective knowledge score, (4) self-rated HIL, and (5) a composite total HIL score integrating all components. The composite score was constructed by first transforming all six HIL-related components to a common 1–4 scale and then computing the arithmetic mean of these standardized components. In addition, a binary high/low HIL variable was created for logistic regression analyses.

Independent variables included demographic factors (age, gender, education, income, population sector, religiosity, and primary language), health-related variables (self-rated health, chronic illness, and medication use), and insurance-related characteristics (insurance type and insurance utilization).

Inferential tests (t-tests, ANOVA, chi-square) were used to assess group differences across demographic and insurance characteristics. To examine predictors of HIL, hierarchical regression analyses were conducted using a four-step modeling strategy. Level 1 included core sociodemographic variables (age, gender, income, and education). Level 2 added population sector and primary language, representing cultural and group-level influences. Level 3 incorporated health-related variables (self-rated health, chronic illness, and medication use). Level 4 introduced insurance characteristics (insurance type and frequency of insurance use), reflecting participants’ direct engagement with the insurance system. This sequential ordering aligns with theoretical models of health insurance behavior, in which demographic characteristics precede cultural context, followed by health-related factors and utilization patterns.

Linear regression models were applied for all continuous outcomes, and logistic regression was used for the binary high/low HIL variable. Model assumptions and diagnostics (normality, homoscedasticity, and multicollinearity checks) were verified for all analyses to ensure robustness. To enhance interpretability, results are reported in two stages: first, unadjusted bivariate analyses provide an initial overview of group-level differences; second, adjusted associations from the multivariable hierarchical regression models identify predictors that remain significant after accounting for potential confounders.

No missing data were detected across survey variables, as completeness checks conducted by the research firm ensured full item responses. Therefore, no imputation or deletion procedures were required.

The study received ethical approval from the Institutional Review Board at Ben-Gurion University. Informed consent was obtained, and data were anonymized to ensure confidentiality.

## Results

### Sample characteristics

The study included 1,012 Israeli residents aged 21 and older, reflecting a diverse cross-section by region, gender (51% women), and age. Most respondents were aged 25–44 (44%), with smaller shares in the 21–24 (8%) and 75+ (5%) brackets.

Ethnically, Israeli-born Jews comprised 72%, Arabs 15%, and foreign-born Jews 13%. Among Jews, 41% were secular, 29% traditional, 8% religious, and 7% ultra-Orthodox. The Arab sample was mostly Muslim (12%), with 2% Christian and 2% Druze.

Education levels were high: 53% held academic degrees and 24% had post-secondary education. Most earned 5,000–15,000 NIS monthly (approx. $1,400–$4,200 USD), compared to the national average of 13,316 NIS ($3,700).

12% earned above 20,000 NIS, and 13% below 5,000 NIS. Employment status showed 70% employed, with smaller proportions retired (12%), unemployed (8%), self-employed (7%), and students (4%). Table 1 details the full demographic breakdown.

**Table 1:** Demographic characteristics of the study population

	*N*	%
**All sample**	1012	100.00%
**Gender**		
Woman	517	51%
Man	495	49%
**Age**		
21–24	84	8%
25–34	232	23%
35–44	217	21%
45–54	170	17%
55–64	150	15%
65–74	111	11%
75+	48	5%
**Sector**		
Israeli born Jews	731	72%
Foreign-born Jews	131	13%
Arab communities	150	15%
**Nationality and Religion**		
Secular Jews	416	41%
Traditional Jews	291	29%
Religious Jews	80	8%
Ultra-Orthodox Jews	75	7%
Muslim Arabs	116	11%
Christian Arabs	17	2%
Druze Arabs	17	2%
**Region of residence**		
Center of Israel	259	26%
Tel-Aviv district	204	20%
North	172	17%
South	145	14%
Haifa district	134	13%
Jerusalem district	75	7%
Judea and Samaria	23	2%
**Family statues**		
Married/Co-habiting with a Partner	700	69%
Single	212	21%
Divorced	68	7%
Widower	17	2%
Refuse to answer	8	1%
Single parent	7	1%
**Education**		
Academic	540	53%
Post-secondary	238	24%
Secondary	216	21%
Primary	18	2%
**Income**		
Over 20 K NIS	119	12%
16–20 K NIS	157	16%
11–15 K NIS	304	30%
5–10 K NIS	304	30%
Up to 5 K NIS	128	13%
**Primary Occupation**		
Employee	704	70%
Retired	125	12%
Unemployed	76	8%
Self-employed	71	7%
Student	36	4%

### HIL levels in the general population

Comprehensive analysis of the HILM revealed nuanced patterns in insurance literacy among the Israeli population. The total mean HILM score of 2.39 (scale 1–4) indicated moderate literacy levels, with notable variations across different components of the measure. Detailed examination of HILM subscales revealed highest competency in HILM subscale 4-behavior in using (2.71), suggesting greater capability in practical insurance utilization. This behavioral score reflects all respondents, although the items describe actions that require prior use of insurance services. Therefore, even participants with relatively limited conceptual knowledge are familiar with navigating insurance benefits when needed. Lower scores were observed in HILM subscale 1 and subscale 3-confidence-related measures (2.32 and 2.29 respectively), indicating potential gaps in self-efficacy regarding insurance decisions. The behavioral domain consistently outperformed the confidence domain (2.48 and 2.31 respectively), revealing a notable disconnect between participants’ actual capabilities and their perceived competence.

**Fig. 1:**
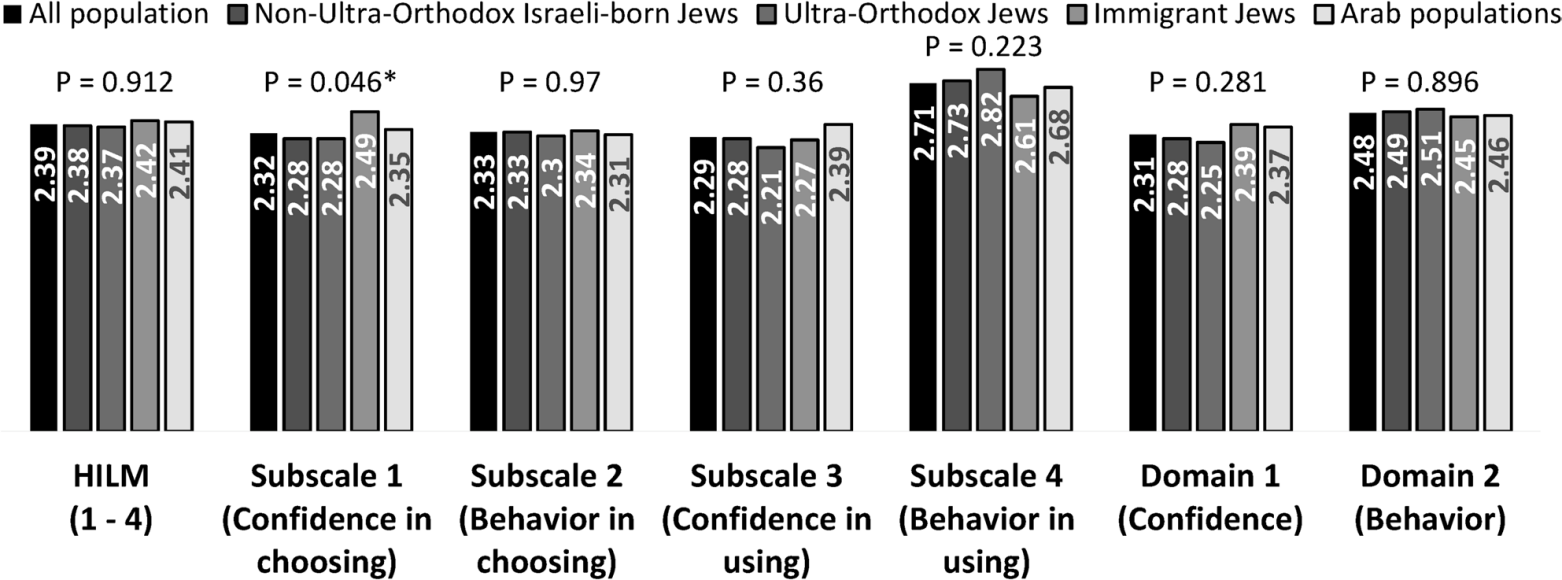
Mean Health Insurance Literacy (HIL) scores across literacy domains. Mean scores of the Israeli population in HILM subscales (knowledge, confidence, behavior) and additional measures (self-reported understanding and objective knowledge). Group differences were assessed using one-way ANOVA. P-values are reported for each HIL component, and asterisks indicate statistically significant differences across groups

**Fig. 2:**
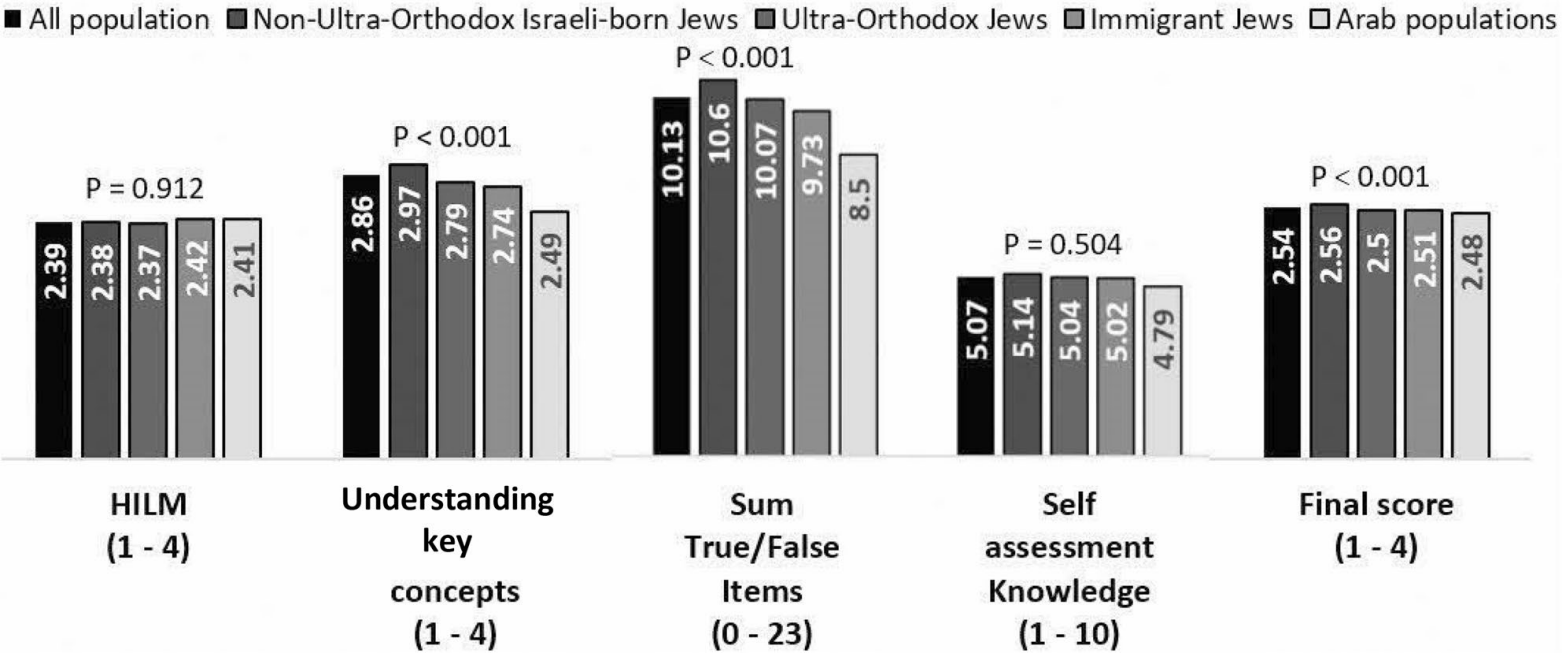
Sectoral differences in Health Insurance Literacy (HIL) components. Mean scores in five HIL components (HILM, familiarity with concepts, objective knowledge, self-assessed knowledge, and total score) across non-Ultra-Orthodox Israeli-born Jews, Ultra-Orthodox Jews, foreign-born Jews, and Arab populations. Group differences were assessed using one-way ANOVA. P-values are reported for each HIL component, and asterisks indicate statistically significant differences across groups.

While self-reported understanding was relatively high (mean = 2.86), objective knowledge scores were lower (44% correct), indicating a gap between individuals’ perceived understanding of health insurance and their objectively measured knowledge. Figures 1 and 2 presents the average scores across the different subscales.

## Demographic variations in HIL

### HIL scores by population sector

Analysis revealed distinct patterns across population sectors. Non-Ultra-Orthodox Israeli-born Jews demonstrated the highest HIL levels, including understanding of insurance concepts (2.97), objective knowledge (10.60/23), and self-assessment (5.14/10). They also scored highly in all HILM subscales, particularly in behavior (2.73).

Ultra-Orthodox Jews showed a unique profile, with moderate total HILM (2.37) but the highest behavioral score (2.82), indicating strong practical skills despite lower conceptual understanding (2.79) and knowledge (10.07). Their self-assessed HIL (5.04) was well-aligned with actual performance.

Foreign-born Jews scored similarly in total HILM (2.42) and showed high confidence in choosing insurance (2.49), though their conceptual (2.74) and objective knowledge (9.73) scores were lower, suggesting overconfidence.

Arab populations had the lowest scores overall, including concept understanding (2.49) and knowledge (8.50), with self-assessment (4.79) consistent with performance. However, they scored relatively well in confidence using insurance (2.39), indicating a potential entry point for targeted interventions. Figures 1 and 2 illustrate these differences across sectors and HIL dimensions.

### Insurance coverage patterns

Analysis of insurance coverage types revealed significant associations with literacy levels. Participants with comprehensive coverage (i.e., those covered by all three insurance tiers, *n* = 419) consistently demonstrated the highest literacy scores across all components. This group achieved significantly higher scores in objective knowledge assessment (mean difference + 2.3 points, *p* < 0.001) and understanding of key insurance concepts (+ 0.4 points, *p* < 0.001) compared to those with basic coverage only.

Those with either supplementary health insurance (*n* = 182) or private commercial insurance (*n* = 67), on top of the public insurance, showed intermediate literacy levels, while participants with basic public insurance only (*n* = 196) demonstrated significantly lower scores across all measures. Most notably, participants uncertain about their insurance type (*n* = 148) consistently showed the lowest score across all components, suggesting that insurance literacy might play a crucial role in awareness and utilization of coverage options.

## Demographic and health factors

understanding of insurance concepts was significantly associated (*p* < 0.05) with all variables. Objective knowledge and self-rated HIL were significantly associated with most variables, while total HIL score showed significance across nearly all, except employment status, country of origin, and health plan membership.

HILM scores differed in pattern, with significance observed only for income, country of origin, health status, insurance type, and insurance utilization. Income and self-reported health status were the strongest and most consistent predictors across all outcomes (*p* < 0.001), followed by insurance type and utilization frequency. Several variables—gender, age, marital and parental status, education, language, and medication use—showed significant associations (*p* < 0.02) with most HIL dimensions, but not HILM. Health plan membership showed limited associations, significant only for understanding of insurance concepts.

Table 2 presents the unadjusted bivariate associations across all HIL components. Regression analyses aligned with these findings: income, health status, and insurance use were robust positive predictors of HILM, objective knowledge, and total score. For total HIL score, higher income (OR = 1.07), better health status (OR = 1.03), and greater insurance use (OR = 1.12) were positively associated, while having only basic coverage was negatively associated (OR = 0.88).

**Table 2:** Associations between demographic characteristics and HIL measure

	**HILM**	**Insurance concepts**	**Insurance knowledge**	**Self-Report HIL**	**Total score**
Gender	*0.312*	0.023*	0.018*	0.025*	0.014*
Age	*0.44*	<0.001*	<0.001*	<0.001*	<0.001*
Marital Status	*0.379*	<0.001*	<0.001*	0.003*	<0.001*
Parental Status	*0.054*	<0.001*	<0.001*	<0.001*	<0.001*
Employment Status	*0.426*	0.015*	*0.055*	*0.205*	*0.086*
Education	*0.121*	<0.001*	<0.001*	0.016*	<0.001*
Income	<0.001*	<0.001*	<0.001*	<0.001*	<0.001*
Primary Language	*0.312*	<0.001*	<0.001*	0.018*	<0.001*
Sector	*0.912*	<0.001*	<0.001*	*0.504*	<0.001*
Religiosity	*0.663*	<0.001*	<0.001*	*0.596*	<0.001*
Country of Origin	0.048*	0.011*	*0.400*	*0.423*	*0.242*
Area of Residence	*0.424*	<0.001*	*0.088*	*0.083*	0.025*
Self-Reported Health Status	0.001*	<0.001*	<0.001*	<0.001*	<0.001*
Chronic Diseases	*0.318*	<0.001*	0.001*	**0.184**	<0.001*
Medications	*0.339*	<0.001*	<0.001*	0.019*	<0.001*
Health plan	*0.743*	<0.001*	*0.062*	**0.158**	*0.05*
Insurance Type	<0.001*	<0.001*	<0.001*	<0.001*	<0.001*
Insurance Use	<0.001*	<0.001*	<0.001*	<0.001*	<0.001*

Introducing additional demographic variables (age, education, marital status, and parental status) modestly improved model performance (R² increased from 28.2% to 29.5%). Age showed a small positive effect; education was marginally negative; other demographic effects were minimal. Further inclusion of medication use, chronic illness, and health plan membership had limited explanatory value, with minimal R² change and mostly non-significant coefficients.

**Fig. 3:**
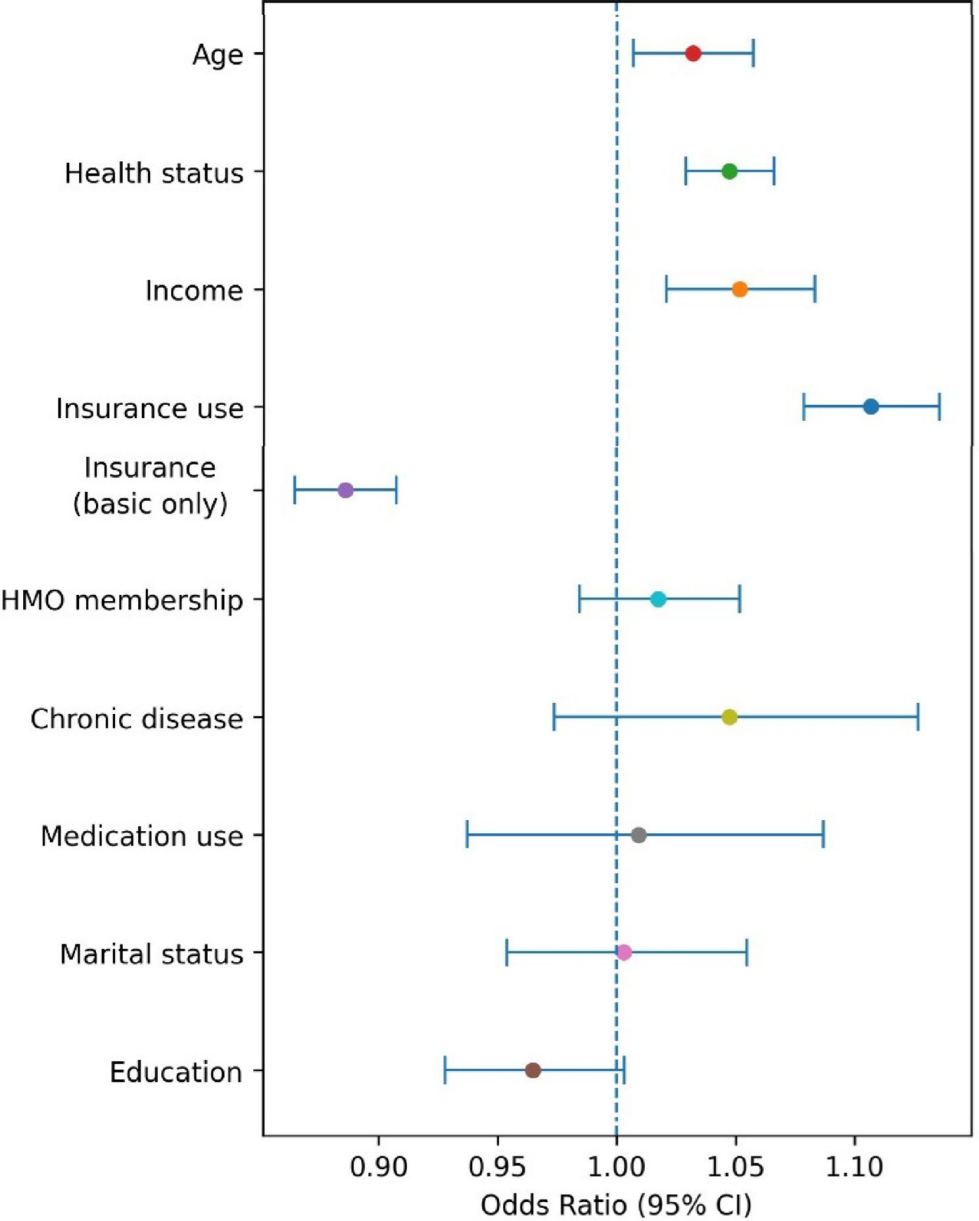
Hierarchical regression results for the final HIL score. The figure presents odds ratios and 95% confidence intervals from the fully adjusted model. Predictors whose confidence intervals do not cross 1.0 are statistically significant. Only statistically significant predictors are interpreted in the text.

## Discussion

This study provides a comprehensive national assessment of HIL in Israel and reveals substantial disparities across population groups. A central finding is the consistent gap between individuals’ conceptual knowledge and their ability to apply this knowledge in real-world decision-making. These results highlight that health insurance literacy is not a uniform construct and that behavioral competencies may lag behind understanding, even within a universal healthcare system. Notably, the observed discrepancy between knowledge and use suggests that interventions focusing solely on information provision may be insufficient, and that practical decision-making support is likely required.

Building on these findings, this study assessed HIL among Israeli adults using a multidimensional, culturally adapted tool. Results revealed moderate overall HIL levels and disparities across sectors, particularly between practical insurance behavior and conceptual understanding. These patterns echo previous findings in both Israeli [14] and international contexts [4], where individuals show greater proficiency in system navigation than in theoretical knowledge.

Compared to international studies, our HILM scores (2.29–2.71) are slightly lower than those in Switzerland [6], but similar to Dutch findings [5]. Notably, we observed smaller gaps between population sectors than reported by Paez et al. in the U.S [4]., with the largest difference found in the behavioral subscale (2.82 among Ultra-Orthodox Jews vs. 2.71 in the general population).

The limited variation across population sectors contrasts with findings from Bardy’s Swiss study [6], which highlighted large disparities between native and non-native speakers. In Israel, foreign-born Jews demonstrated HIL levels comparable to Israeli-born Jews, suggesting that factors like education, socioeconomic status, and cultural attitudes may be more influential than language.

Our sectoral analysis aligns with previous research. Higher HIL among non-Ultra-Orthodox Jews is consistent with Levin-Zamir et al. [11], while the strong behavioral scores among Ultra-Orthodox Jews reflect findings from Arbel et al. [21] on community-based knowledge-sharing. Arab communities showed consistently lower scores, similar to patterns noted by Green et al. [14], Baron-Epel et al. [22], and Ali et al. [23], although their relatively high score in confidence (HILM subscale 3) suggests potential for focused interventions, as supported by Shibli et al. [24].

When situated within the broader literature on literacy, the patterns observed in this study both align with and diverge from findings in other domains [15, 16]. Similar to research on general and health literacy, lower HIL levels were observed among Arab populations and individuals with lower income, reflecting structural and informational inequalities documented in previous Israeli and international studies [14]. At the same time, the relatively modest role of formal education and the strong performance in behavioral domains suggest that HIL may be shaped more by experiential exposure and system interaction than by traditional cognitive literacy alone. Similar patterns have been reported in previous studies, which found that frequent engagement with health insurance systems and direct experience using coverage were more strongly associated with functional insurance literacy than formal educational attainment [5, 25].

These findings indicate that while HIL shares common social gradients with other forms of literacy, it also reflects system-specific demands. In the Israeli context, automatic enrollment in national health insurance and widespread use of supplementary coverage may enable functional navigation of insurance services even in the absence of strong conceptual understanding. This distinction underscores the importance of examining HIL as a related, but not interchangeable, construct within the broader literacy landscape.

Insurance coverage patterns correlated strongly with HIL levels. Participants with all three tiers of coverage demonstrated higher scores across dimensions, supporting findings by Park et al. [26] and aligning with broader evidence linking voluntary insurance with improved outcomes, such as the European study by Uejima et al. [27].

Regression analyses confirmed the positive effects of income and self-reported health status, as seen in studies by Paez et al. [1], Liu et al. [28], and Merrell et al. [29]. The limited role of education in predicting HIL, however, contrasts with past research [30–32]. This discrepancy may reflect the complexity of insurance systems, where hands-on experience often outweighs formal education, as suggested by Chen and Lee [31], Brown et al. [32], and Call et al. [25].

The moderate explanatory power of the regression models (R² ≈ 30%) suggests that, beyond the socioeconomic, health-related, and insurance-related variables included in this study, additional factors are likely to shape health insurance literacy. These may include digital literacy, institutional trust, and informal community-based mediation, which have been shown in previous research to influence individuals’ ability to navigate complex health systems. In contexts such as Israel, where insurance arrangements are multifaceted and information is often accessed through both formal and informal channels, these factors may play a particularly important role.

In this regard, social capital may represent a key explanatory mechanism linking individual characteristics and system navigation. Prior research has demonstrated that trust in community institutions and participation in local networks can significantly influence insurance-related decision-making and engagement. For example, Ko et al. [33] found that higher levels of social capital were associated with increased willingness to enroll in community-based health insurance schemes in Nepal, highlighting the potential relevance of community-mediated knowledge and trust-based pathways for understanding HIL beyond individual-level determinants.

An important pattern emerging from the data is the discrepancy between participants’ relatively high behavioral scores, which reflect frequent use of insurance services and familiarity with navigating coverage in practice, and their substantially lower objective knowledge scores. This pattern may reflect the structure of the Israeli system, in which individuals are automatically covered by the national benefits package and many also hold supplementary or private insurance by default. As a result, people can use insurance benefits effectively without necessarily possessing a strong conceptual understanding of insurance terminology, coverage rules, or entitlements. Similar gaps between high utilization and limited knowledge have also been noted in previous studies examining consumer insurance behavior in accessible or multi-layered insurance systems (Edward et al., 2019; Nobles et al., 2019). This finding highlights the need to strengthen foundational knowledge, even in contexts where practical access is relatively seamless.

While education traditionally correlates with health knowledge [1, 34, 35], our findings suggest that in the Israeli context, factors such as practical experience [36], field of study [37], or direct exposure to the health system [38] may better explain variations in HIL. Israel’s universal health coverage may reduce the incentive for active engagement with insurance systems, unlike in countries with more fragmented coverage models, where health insurance literacy plays a crucial role in the process and outcomes of choosing insurance plans [39].

These findings expand our understanding of HIL by demonstrating how different dimensions - confidence, behavior, knowledge, and self-assessment - interact within specific cultural and systemic contexts. Although general literacy gaps are well-documented [1–3], this study highlights how they vary across demographic lines. However, several limitations should be acknowledged. The cross-sectional design precludes causal inference, and the use of self-reported online data may have introduced reporting and social desirability biases. In particular, respondents may overestimate their understanding of or engagement with health insurance, potentially inflating subjective measures relative to objectively assessed knowledge.

Although the sample was stratified to reflect key demographic characteristics of the Israeli adult population, some subgroups were relatively small, potentially limiting generalizability. In addition, the use of an online, panel-based survey may have constrained representativeness among populations with lower internet access, a concern that is particularly relevant for ultra-Orthodox and Arab populations. Respondents from these groups may therefore represent more digitally connected individuals who are also more engaged with formal institutions than the broader populations within these sectors. More broadly, reliance on an online survey may have resulted in the overrepresentation of individuals with higher levels of digital literacy, health system engagement, and general informational access. As a result, the overall HIL levels observed in this study may represent an upper-bound estimate of population-level health insurance literacy in Israel.

Furthermore, the panel-based survey design precluded calculation of a traditional response rate, limiting the ability to assess non-response bias. While this approach is widely used in national surveys in Israel, it nonetheless constrains the extent to which full population representativeness can be claimed. Moreover, the adapted knowledge measure, while carefully developed, pilot-tested, and reviewed for content validity, has not yet undergone external validation. Future studies should seek to externally validate this measure across different populations and settings, as well as to incorporate sensitivity analyses and more detailed subgroup analyses, to further strengthen the robustness and interpretability of findings.

Given the use of an online panel, the sample may underrepresent individuals with limited digital access or lower technological literacy. As a result, overall levels of health insurance literacy may be overestimated, particularly among more digitally connected populations.The survey took place during an active phase of the 2024 Israel–Hamas war. This context may have influenced participation patterns, particularly among individuals experiencing displacement, reserve duty, or heightened stress. Although sampling quotas were fully met, certain groups may have been underrepresented, and response behavior may have been affected by the wartime environment. These potential biases are discussed in the study limitations. Future studies should adopt longitudinal designs to track HIL changes and incorporate qualitative methods to explore underlying drivers of disparity. Qualitative approaches, as recommended by Quiroga [20], can provide richer insights into how individuals from different communities navigate complex insurance structures. This may include examining the influence of local leadership or communal knowledge channels, as suggested by Arbel et al. [21]. Despite these limitations, the study offers novel insights into HIL patterns within a universal healthcare system.

Further research should investigate the role of academic disciplines in shaping HIL, and the relationship between perceived literacy and actual knowledge. In line with past work [40, 41], policymakers should promote culturally tailored educational programs, especially among Arab and foreign-born populations. Resources should be simplified, translated, and widely accessible. Integrating HIL into school curricula and adult education may enhance awareness and improve navigation of insurance systems. Regular monitoring of HIL can support the design of effective interventions.

Finally, our findings hold relevance beyond Israel. Minority and migrant populations in many countries face similar challenges. The comprehensive, culturally sensitive assessment approach demonstrated here may be applied globally to address inequities in insurance comprehension. As shown by Guerrero et al. [38], underserved groups benefit from tools that bridge structural and cognitive gaps in insurance access.

## Conclusion

This study underscores the multifaceted nature of HIL and its critical role in enabling individuals to navigate complex healthcare systems effectively. The findings reveal moderate total HIL levels among the Israeli population, with pronounced disparities across population groups. Gaps among Arab and foreign-born communities highlight systemic barriers, underscoring the need for targeted education and policy reforms to improve healthcare equity. Additionally, this study emphasizes the critical role of financial and health determinants in shaping HIL scores, while suggesting that demographic and additional health-related variables have a more nuanced or limited influence.

These findings carry important implications for policy-making in Israel. They offer the first comprehensive, population-based evidence on health insurance literacy, providing a foundation for designing targeted strategies to strengthen public understanding of insurance concepts. By identifying concrete gaps in knowledge, confidence, and utilization, this study highlights key entry points for policy interventions that could enhance informed decision-making, promote more equitable use of insurance resources, and support future reforms to Israel’s evolving multi-layered insurance system.

## Supplementary Information

Supplementary Material 1

## Data Availability

The datasets generated and/or analyzed during the current study are available from the corresponding author on reasonable request.

## Abbreviations

HIL: Health insurance literacy
HILM: Health insurance literacy measure
IRB: Institutional review board
ANOVA: Analysis of variance
OR: Odds ratio
CI: Confidence interval
USD: United States dollar
NIS: New Israeli shekel

## Declarations

### Use of AI in manuscript preparation

During the preparation of this work, the authors used ChatGPT and Perplexity AI tools for language editing and improving readability. After using these tools, the authors reviewed and edited the content as needed and take full responsibility for the final version of the manuscript.
