# Supplementary material for: Health insurance literacy in Israel: gaps between knowledge and use in a universal healthcare system
Source: Isr J Health Policy Res. 2026 Apr 16;15:13. doi: 10.1186/s13584-026-00759-y (PMC13085595; doi:10.1186/s13584-026-00759-y)
Supplement: Supplementary file 1 — Supplementary Material 1 [file 13584_2026_759_MOESM1_ESM.docx]

# **Israeli Health Insurance Literacy Questionnaire**

## **Chapter One - Skills and Capabilities in Using Health Insurance**

(Translation and Adaptation of HILM Questionnaire)

**How confident or not confident are you ...**

|  | Not at all sure | Somewhat sure | Fairly sure | Very  sure |
| --- | --- | --- | --- | --- |
| 1. You understand the concepts related to health insurance in Israel |  |  |  |  |
| 1. You understand the differences between HMO supplementary insurance and private commercial health insurance |  |  |  |  |
| 1. You can estimate your monthly insurance cost for next year (excluding emergencies) |  |  |  |  |
| 1. You know what questions to ask to choose the right health insurance for you and your family |  |  |  |  |
| 1. You know where to find the information needed to choose the right health insurance for you and your family |  |  |  |  |
| 1. You will succeed in choosing the most suitable health insurance for you or your family at the HMO or with a commercial insurance company |  |  |  |  |

**When you want to purchase a new health insurance plan and you're comparing different health insurance plans, how likely are you to understand...**

|  | Not at all likely | Somewhat likely | Quite likely | Very likely |
| --- | --- | --- | --- | --- |
| 1. If the insurance policy covers unexpected costs, such as rehabilitation after an accident or stroke |  |  |  |  |
| 1. How much you'll need to pay for private hospital surgery |  |  |  |  |
| 1. How much you'll need to pay out of pocket for visiting a specialist not through the HMO |  |  |  |  |
| 1. How much you'll need to pay for prescription medications not in the national health basket |  |  |  |  |
| 1. Which doctors and hospitals are covered by the insurance policy |  |  |  |  |
| 1. What are the differences between various insurance policies offered to you |  |  |  |  |

**When using or activating your health insurance at HMOs/private insurance company, and before receiving certain treatment, how confident do you feel that...**

|  | Not at all sure | Somewhat sure | Fairly sure | Very  sure |
| --- | --- | --- | --- | --- |
| 1. You'll know how to check what is and isn't covered by your health insurance before receiving treatment |  |  |  |  |
| 1. You'll know what to do if the insurer refuses to pay or reimburse for treatment you think should be covered |  |  |  |  |
| 1. You'll succeed in understanding the cost-sharing between out-of-pocket payment and insurance coverage |  |  |  |  |
| 1. You'll know what questions to ask if you have a problem with coverage or reimbursement |  |  |  |  |
| 1. You'll know most details required to use your health insurance |  |  |  |  |

**Please indicate how likely are you to act in the following ways when using or activating your health insurance at HMOs/private insurance company before receiving certain treatment...**

|  | Not at all likely | Somewhat likely | Quite likely | Very likely |
| --- | --- | --- | --- | --- |
| 1. Contact customer service of the HMO and/or private insurance company to ask which treatment is covered by your health insurance |  |  |  |  |
| 1. Succeed in understanding what is and isn't covered by your insurance policy before certain treatment |  |  |  |  |
| 1. Check if certain treatment or doctor is included in your insurance policy before starting treatment |  |  |  |  |
| 1. Read and check the quarterly bill you receive from the HMO or private insurance company |  |  |  |  |

## **Chapter Two - Familiarity with Concepts in the Israeli Health Insurance Market**

**For the following concepts in health insurance, please indicate what applies to you:**

|  | Never heard of this concept | Heard of the concept but don't understand it | Know the concept and understand it in context, but can't explain it | Understand it well and can explain its meaning |
| --- | --- | --- | --- | --- |
| 1. National Health Insurance Law |  |  |  |  |
| 1. Supplementary Health Insurance |  |  |  |  |
| 1. Health Declaration |  |  |  |  |
| 1. Qualification Period |  |  |  |  |
| 1. Deductible/Copayment |  |  |  |  |
| 1. Actuary |  |  |  |  |
| 1. Underwriting |  |  |  |  |
| 1. Pre-existing Condition |  |  |  |  |
| 1. Premium |  |  |  |  |
| 1. Changing Premium |  |  |  |  |
| 1. Health Basket |  |  |  |  |

## **Chapter Three - True/False Knowledge Questions**

Please note:

- "Supplementary Health Insurance" refers to additional health services in HMOs (like Clalit Mushlam and Platinum, Maccabi Gold and Silver and Maccabi Sheli, etc.)
- "Private-Commercial Health Insurance" refers to commercial insurance (private or group) from insurance companies like Harel, AIG, Migdal, Menora, etc.

**For each statement, please indicate if it is True or False regarding the current Israeli healthcare system:**

|  | True | False | Don't Know |
| --- | --- | --- | --- |
| 1. Public-national health insurance is funded by health insurance tax (collected by National Insurance Institute) and supplemented by state budget sources |  |  |  |
| 1. In addition to health tax, payment for national (public) health insurance is paid monthly directly by the insured to the HMO |  |  |  |
| 1. Payment for supplementary health insurance is made according to usage only |  |  |  |
| 1. Payment for supplementary health insurance is made according to a fixed monthly premium plus copayment for services used |  |  |  |
| 1. The HMO may refuse to provide health services included in the service basket to a member who hasn't paid or is late for insurance payments |  |  |  |
| 1. Every Israeli resident is legally entitled to receive any treatment or medication included in the national-public health basket (based on medical need) |  |  |  |
| 1. A person who doesn't pay monthly payments to the HMO can visit a family doctor for free or for a low quarterly or one-time copayment |  |  |  |
| 1. A person who doesn't pay directly to the HMO monthly can seek additional consultation (second opinion) with a specialist for free or for a low quarterly or one-time copayment |  |  |  |
| 1. Arriving at the ER without professional referral is free or costs a low quarterly or one-time copayment |  |  |  |
| 1. Surgery in a private hospital allowing a choice of surgeon is included in public insurance rights |  |  |  |
| 1. Child development treatments are only given to those with supplementary or private insurance |  |  |  |
| 1. Fertility treatments are only given to those with supplementary or private insurance |  |  |  |
| 1. Alternative treatments (complementary medicine) are only given to those with supplementary or private insurance |  |  |  |
| 1. To purchase national-public insurance, you must sign a health declaration |  |  |  |
| 1. To purchase supplementary insurance, you must sign a health declaration |  |  |  |
| 1. To purchase private-commercial insurance, you must sign a health declaration |  |  |  |
| 1. In supplementary insurance, the HMO can decide to terminate the insurance agreement on its own initiative |  |  |  |
| 1. In private-commercial health insurance, the insurance company can decide to terminate the insurance agreement on its own initiative |  |  |  |
| 1. In supplementary insurance, the HMO can decide not to insure a person suffering from a chronic illness or above a certain age |  |  |  |
| 1. In private-commercial health insurance, the insurance company can decide not to insure a person suffering from a chronic illness or above a certain age |  |  |  |
| 1. In national-public health insurance, using some insurance coverages requires waiting for a qualification/waiting period from policy purchase |  |  |  |
| 1. In supplementary insurance, using some insurance coverages requires waiting for a qualification/waiting period from policy purchase |  |  |  |
| 1. In private-commercial health insurance, using some insurance coverages requires waiting for a qualification/waiting period from policy purchase |  |  |  |

## **Chapter Four - Self-Assessment**

1. To what extent do you feel you understand or don't understand health insurance and the health insurance market in Israel? On a scale of 1 - "Don't understand at all" to 10 - "Understand excellently"

## **Chapter Five - Demographic Questions + Insurance Use**

1. Gender
2. Birth year
3. Sector/Religion
4. Level of religiosity
5. Marital status
6. Children: Yes or No
7. Living area
8. Education
9. Income
10. Country if birth and immigration year
11. Employment status
12. What is the main language you speak at home today?
13. Please rate your health condition relative to your age between 1-10 where 1 means "not good at all" and 10 means "very good"
14. Do you have any chronic illnesses? - Yes / No
15. Do you take medications regularly? - Yes / No
16. Which HMO are you insured with?
17. What health insurance do you have?
18. Have you ever used the benefits in supplementary insurance?
19. Have you ever used/requested reimbursement from your private-commercial health insurance?
